# A Dual-Band Eight-Element MIMO Antenna Array for Future Ultrathin Mobile Terminals

**DOI:** 10.3390/mi13081267

**Published:** 2022-08-06

**Authors:** Chuanba Zhang, Zhuoni Chen, Xiaojing Shi, Qichao Yang, Guiting Dong, Xuanhe Wei, Gui Liu

**Affiliations:** College of Electrical and Electronic Engineering, Wenzhou University, Wenzhou 325035, China

**Keywords:** 5G, multiple input–multiple output (MIMO), ultrathin, smartphone, dual-band

## Abstract

An ultrathin dual-band eight-element multiple input–multiple output (MIMO) antenna operating in fifth-generation (5G) 3.4–3.6 GHz and 4.8–5 GHz frequency bands for future ultrathin smartphones is proposed in this paper. The size of a single antenna unit is 9 × 4.2 mm^2^ (0.105 λ × 0.05 λ, λ equals the free-space wavelength of 3.5 GHz). Eight antenna units are structured symmetrically along with two sideboards. Two decoupling branches (DB1 and DB2) are employed to weaken the mutual coupling between Ant. 1 and Ant. 2 and between Ant. 2 and Ant. 3, respectively. The measured −10 dB impedance bands are 3.38–3.82 GHz and 4.75–5.13 GHz, which can entirely contain the desired bands. Measured isolation larger than 14.5 dB and 15 dB is obtained in the first and second resonant modes, respectively. Remarkable consistency between the simulated and measured results can be achieved. Several indicators, such as the envelope correlation coefficient (ECC), diversity gain (DG), total active reflection coefficient (TARC), and multiplexing efficiency (ME), have been presented to assess the MIMO performance of the designed antenna.

## 1. Introduction

Fifth-generation (5G) communication is burgeoning, demanding wireless devices with a transmission data rate as high as possible. Multiple input–multiple output (MIMO) technology possesses promising application prospects in improving the data rate. Recently, many sub-6 GHz 5G smartphone MIMO antennas have been developed [1,2,3,4,5,6,7,8,9,10,11,12,13,14,15,16], such as four-element MIMO antennas [2,3,4,5], eight-port smartphone antennas [6,7,8,9,10], and even twelve-element MIMO antennas [11,12]. One nonnegligible challenge encountered during the design process is the method to effectively weaken the mutual electromagnetic coupling between antenna elements in a MIMO antenna array. However, numerous decoupling mechanisms have been put forward, such as polarization diversity [9], defected ground structure (DGS) [12,13], decoupling branches [14], neutralization lines [15], and orthogonal mode [16]. More attention still needs to be focused on the decoupling design in the MIMO antenna array.

The usual height of the lateral side frame of a conventional smartphone’s antenna [2,3,6,7] is 7 mm, which is not conducive to implementing future ultrathin smartphones. Some low-profile MIMO antennas for the 5G handsets have been proposed recently. In [17], a compact four-element MIMO antenna pair for 5G mobile was presented, integrating two antenna elements at a close distance of 1.2 mm. The designed antenna pair resonated precisely at 3.5 GHz. One more worthy mention is that the overall volume of the MIMO system was 150 × 73 × 6 mm^3^, which realized a 1 mm reduction in the height of the lateral side frame. Another self-decoupled four-element antenna pair [18] functioning in the 3.5 GHz band (3.4–3.6 GHz) with the same height of 6 mm has been presented, and the mutual coupling of the antenna pair was decreased to 16.5 dB. In [19], a low-profile, high-isolation eight-port MIMO antenna for the 5G handset was presented, and the height of the lateral sideboard was 5.3 mm.

This paper presents an ultrathin eight-port MIMO antenna working at 5G 3.4–3.6 GHz and 4.8–5 GHz frequency bands. The integral volume of the proposed antenna is only 145 × 70 × 5 mm^3^, which is thinner than other published 5G smartphone antennas. Eight antenna elements are manufactured along the inner face of two sideboards. Two decoupling branches (DB1 and DB2) are employed to attenuate the mutual coupling. The proposed antenna is fabricated and measured. The measured −10 dB impedance bands are 3.38–3.82 GHz and 4.75–5.13 GHz, which can fully contain two target bands. A measured lowest isolation (14.5 dB) emerged in S_23_ around 3.5 GHz. DHM mode is provided to assess practical application ability. ECC, DG, TARC, and ME are calculated to evaluate diversity performance.

## 2. Antenna Structure

**Figure 1:**
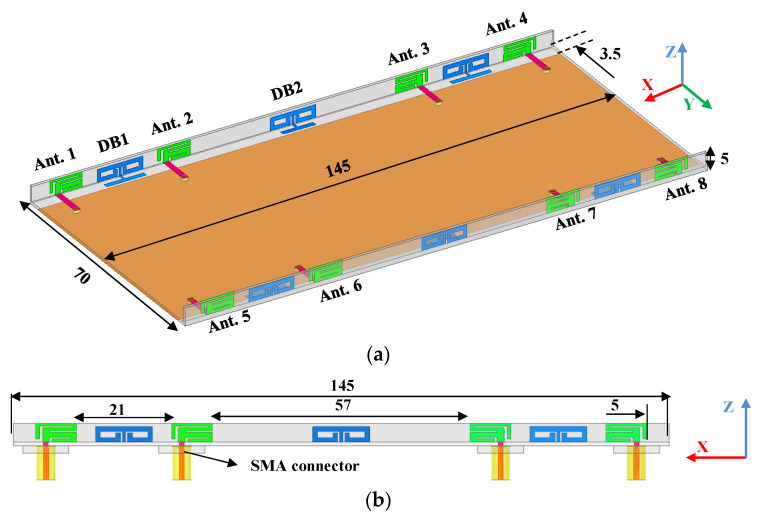

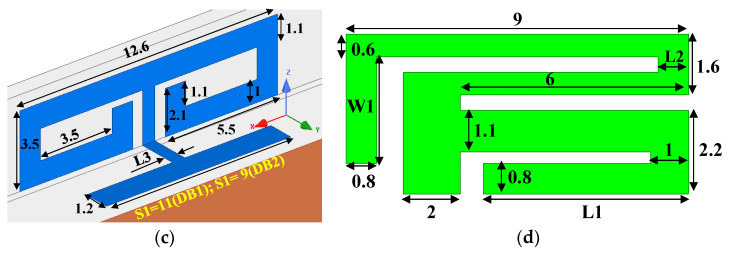
Perspectives of the proposed antenna. (**a**) Overall view, (**b**) side view, (**c**) dimensions of DB1 and DB2, and (**d**) detailed structure of an antenna element. (All values are in millimeters).

The overall view and lateral perspective of the proposed antenna array are shown in Figure 1. Eight antenna elements are printed along the inner side of two sideboards with a size of 145 × 4.2 × 0.8 mm^3^, which are constructed perpendicularly to the system board. The size of the system board is 145 × 70 × 0.8 mm^3^. The sideboards and system board substrate are an FR4 substrate with loss tangent = 0.02 and relative permittivity = 4.4. The height of the whole smartphone is only 5 mm, since the sideboards are placed on the system board. A 2 mm-wide microstrip line feeds each element through an SMA connector via the hole from the bottom of the system board. The designed DBs are separately printed on the inner face and upper side of the sideboard and system board, which are welded together. A ground plane (145 × 70 mm^2^) with two rectangular ground clearances (145 × 3.5 mm^2^) is fabricated on the bottom of the system board. The dimensions of DB1(2) and the detailed construction of a fundamental antenna element are illustrated in Figure 1c,d. Parameters that affect antenna performance are described as variables rather than a fixed value. Notably, the values of *S1* of DB1 and DB2 are 11 mm and 9 mm, respectively.

## 3. Working Mechanism and Application Scenario

In this section, the design evolution steps are first presented to understand the operating mechanism better. Consequently, the role of the DBs is analyzed. The third part shows the current vector distribution of the proposed antenna at 3.5 GHz and 4.9 GHz when DB1 is utilized or not, and some variables are selected to be analyzed. The last portion of this section provides a scenario where this device is held in dual-hand mode (DHM).

### 3.1. Design Procedure

**Figure 2:**
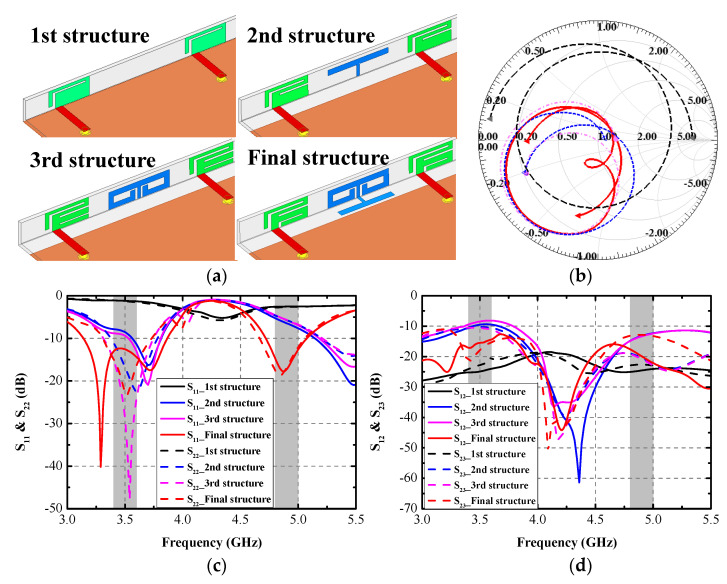
(**a**) Design evolution, (**b**) simulated Smith chart of S_11_, (**c**) simulated S_11_ and S_22_, (**d**) simulated S_12_ and S_23_.

This section presents a precise design evaluation of the proposed antenna. Figure 2a gives the four structures during the design process. The first structure is a single rectangular plane with a small open-ended L-shaped slot. It can be seen from Figure 2c that an obvious resonant mode around 4.3 GHz of Ant. 1 and Ant. 2 is obtained. However, the port impedance matching needs to be optimized. As shown in Figure 2b, the simulated normalized port impedance curve is far away from the center point of the Smith chart. Another small rectangular slot and ground clearance (145 × 3.5 mm^2^) are cut from the antenna element and grounding plane in the second structure. A T-shaped strip is introduced to diminish the mutual coupling between Ant. 1 and Ant. 2. It can be distinctly observed from Figure 2c that two resonant modes (around 3.6 GHz and 5.5 GHz) are excited. Little frequency offset between S_11_ and S_22_ occurs because of the two elements’ different locations. The mutual coupling S_12_ of the second structure is 10 dB and 13 dB in the lower and higher bands, respectively.

Furthermore, a significant enhancement in the normalized impedance matching condition is acquired, which can be observed in Figure 2b. In the third structure, a small rectangular slot is cut from the antenna element of the second structure. An extra two C-shaped strips are added to the upper terminals of the aforementioned T-shaped decoupling branch. The simulated S_22_ obtains a good matching condition at 3.53 GHz. The lowest isolation of 8 dB occurs at 3.53 GHz, and little isolation promotion at 5.5 GHz is realized. The final antenna element structure is produced by cutting a small rectangular slot in the feedline side of the lowest rectangular strip and reconnecting it on the other side. At the same time, another newly introduced horizontal T-shaped branch is connected to the existing decoupling branch as the third structure. The final design of an antenna element and DB1 are generated in Figure 2a. The simulated S_11_ and S_22_ can entirely contain the target bands, and the isolation S_21_ is also lifted to 15 dB and 18 dB across 3.4–3.6 GHz and 4.8–5 GHz, respectively. Table 1 lists the simulated normalized impedance at each design stage at 3.5 GHz and 4.9 GHz. The final structure obtains better impedance matching performance than the former three design stages.

### 3.2. Study of the Role of the DBs

**Figure 3:**
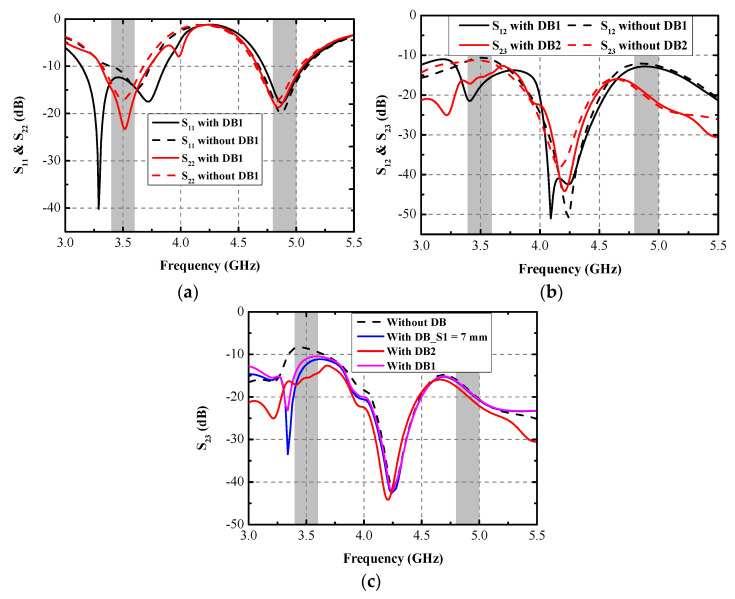
Simulated (**a**) S_11_ and S_22_ and (**b**) S_12/23_ with/without DBs, (**c**) S_23_ with various values of *S1*.

**Figure 4:**
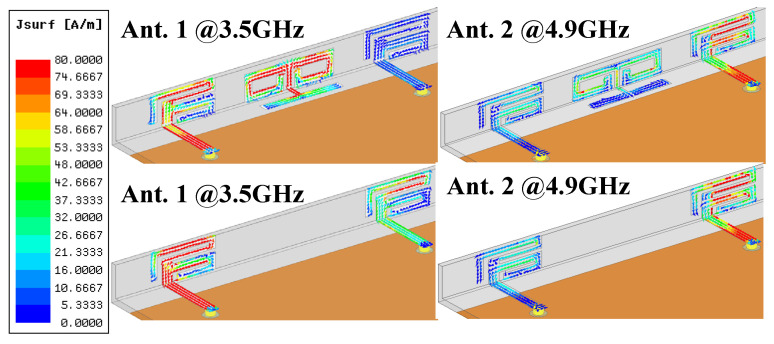
Current distribution at two resonant modes when DB1 is utilized or not.

This section presents the simulated results with/without DBs. As shown in Figure 3a, when there is no DB1, the simulated S_11_ and S_22_ can still cover the desired bands. However, the first resonant frequency of Ant. 1 moves to 3.6 GHz, while the other operation band causes little influence. Figure 3b illustrates the simulated isolation curves S_12_ and S_23_ with/without DB1 and DB2. The utilization of the DBs can effectively attenuate the mutual coupling at 3.5 GHz, while there is little impact on the mutual coupling at 4.9 GHz, as shown in Figure 4. The worst simulated isolation (14 dB) appeared at S_12_. Figure 3c portrays the simulated S_23_ with various values of *S1*. Relatively low isolation (8 dB) at 3.5 GHz was obtained when no DB was used. After the DB1 is constructed between Ant. 2 and Ant. 3, a distinctly improving trend occurred to S_23_, as depicted in Figure 3c, but it was still insufficient. By adjusting the length of *S1*, isolation performance can be improved. When the value of *S1* is 9 mm, the simulated S_23_ satisfies the requirement of 15 dB within the desired bands at 3.5 GHz. When the value of *S1* decreases to 7 mm, some deterioration happens to S_23_, as shown in Figure 3c. The final optimized value of *S1* is 9 mm.

### 3.3. Current Distribution and Parametric Analysis

The simulated current distribution of Ant. 1 and Ant. 2 at two operating frequencies when the DB1 is adopted or not are portrayed in Figure 4. When Ant. 1 is excited at 3.5 GHz, the strongest current density is allocated over the upper L-shaped slot of Ant. 1 and the inner edges of slots of the lateral section of DB1. The introduction of DB1 significantly decreases the current density coupled in Ant. 2 when Ant. 1 is excited at 3.5 GHz. When Ant. 2 is excited at 4.9 GHz, the maximum current spread around the middle rectangular slot of Ant. 2. There is no significant difference in the coupling current of distribution of Ant. 1 when DB1 is applied or not. The utilization of DB1 powerfully absorbs the mutual magnetic coupling existing between Ant. 1 and Ant. 2 at 3.5 GHz, hence enhancing the isolation. The slight improvement resulting from the DB1 is realized upon S_12_ at 4.9 GHz, which is also consistent with the simulated curves in Figure 3b.

**Figure 5:**
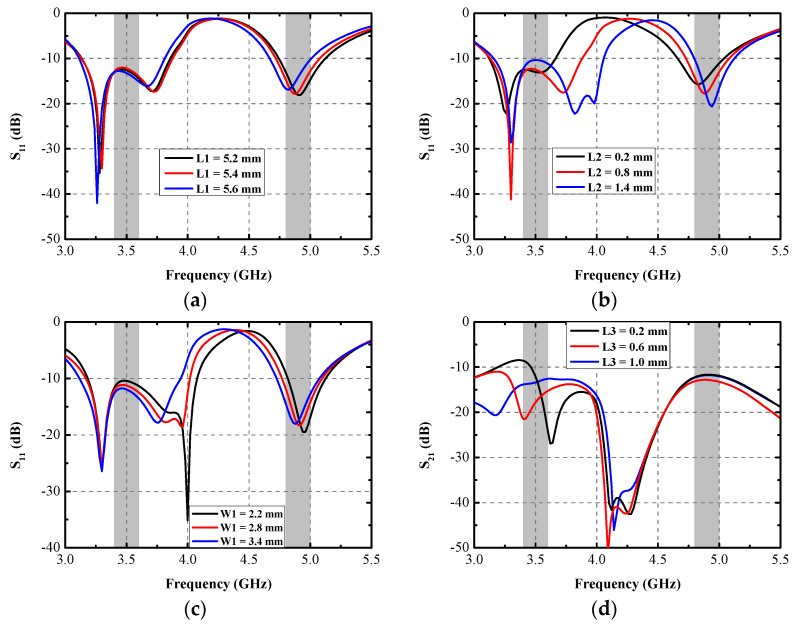
Simulated S_11_ with various (**a**) *L1*, (**b**) *L2*, (**c**) *W1*, and (**d**) S_21_ with different *L3*.

According to the current distribution, numerous parameters are selected to make a parametric analysis, as shown in Figure 5. Little resonant frequency offset of the latter band arises with the increase in *L1*, and almost no impact is caused on the first operating band. The final value of *L1* is 5.4 mm. As illustrated in Figure 5b, the variation in *L2* affects all three resonant points. With the increase in *L2*, the impedance matching condition at 3.5 GHz deteriorates, the middle resonant mode around 3.7 GHz shifts to the higher frequency, and the ultimately optimized length of *L2* is 0.8 mm. Without influencing the first resonant mode of Ant. 1, the addition of the value of *W1* contributes a lot to the movement of the other two resonant modes. As illustrated in Figure 5c, the second resonant mode moves to 4 GHz when the value of *W1* is 2.2 mm, and the final impedance band is not satisfied. The ultimately modified value of *W1* is 2.8 mm. Parameter *L3* mainly affects the decoupling performance. A significant difference in the isolation at 3.5 GHz occurs with the varying of *L3*. When the value of *L3* equals 0.6 mm, the simulated S_21_ is separately larger than 15 dB and 14 dB at 3.5 GHz and 4.9 GHz, respectively.

### 3.4. Application Scenario

**Figure 6:**
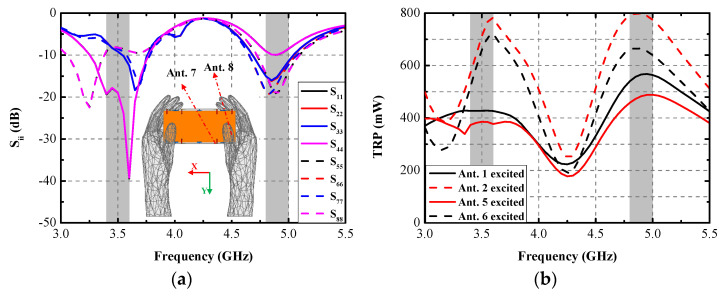
Simulated (**a**) S-parameters and (**b**) TRPs for the presented antenna held in DHM.

**Figure 7:**
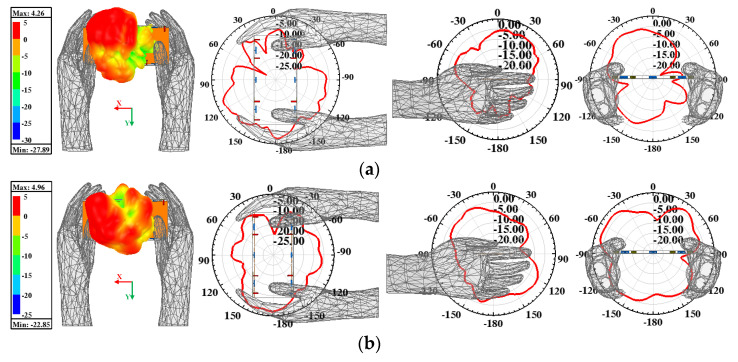
Simulated 3D and 2D patterns when (**a**) Ant. 8 excited at 3.5 GHz and (**b**) Ant. 7 excited at 4.9 GHz.

**Figure 8:**
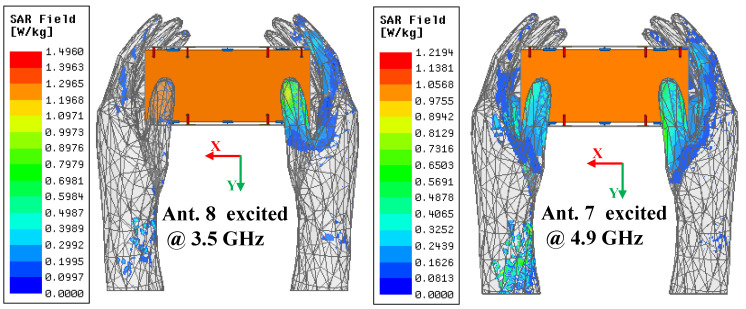
SAR field distribution.

An application scenario of the presented antenna held in dual-hand mode (DHM) is studied to identify the robustness and practicability of the proposed MIMO antenna array. Figure 6 plots the simulated S-parameters in DHM and the −10 dB bandwidth of Ant. 5, with Ant. 8 not being able cover two target bands. The −10 dB impedance matched bandwidth of Ant. 2, Ant. 3, Ant. 6, and Ant. 7 can contain 5G 3.6–3.8 GHz and 4.8–5 GHz frequency bands. The simulated S_11_ and S_44_ can still wholly cover the two desired bands. Figure 6b provides the total radiated power (TRP) of the proposed antenna when Ant. 1, Ant. 2, Ant. 5, and Ant. 6 are separately excited with 1 W input power. The radiating ability of four inner elements (Ant. 2, Ant. 3, Ant. 6, and Ant. 7) are generally better than the other four elements constructed in the corners of the system substrate, which have the closest distance to the hand tissue compared with the inner four elements. Figure 7 presents the proposed antenna’s simulated three-dimension (3D) and two-dimension (2D) radiation patterns when Ant. 8 and Ant. 7 are independently excited at 3.5 GHz and 4.9 GHz, respectively. The simulated specific absorption rate (SAR) distribution when Ant. 8 and Ant. 7 are separately excited with 100 mW input power at two resonant modes, as shown in Figure 8. A maximum SAR value of 1.45 W/kg and 1.22 W/kg is acquired at 3.5 GHz and 4.9 GHz, respectively. Both SAR values are lower than the European and American requirements of 2.0 W/kg and 1.6 W/kg.

## 4. Experimental Results

**Figure 9:**
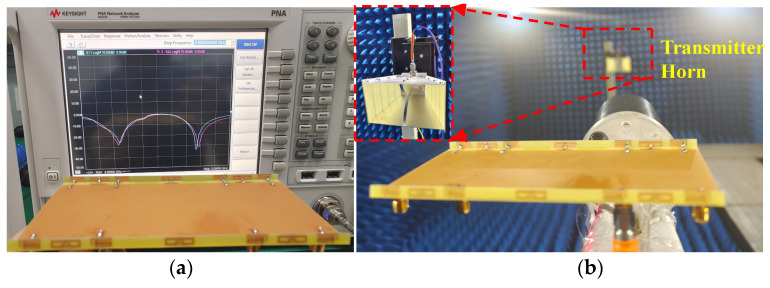
Photograph of the manufactured model of the proposed antenna and the experimental environment (**a**) measured by the VNA and (**b**) measured in the anechoic chamber.

**Figure 10:**
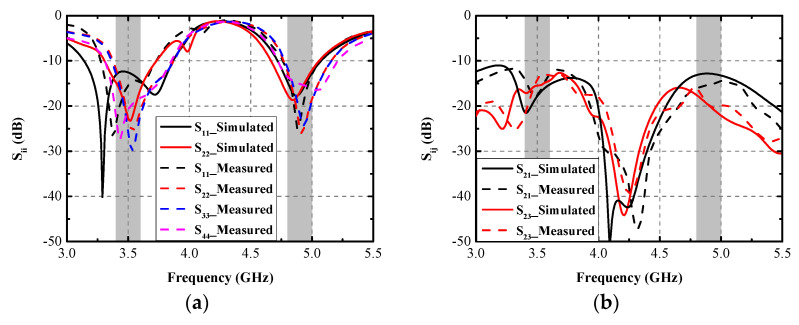

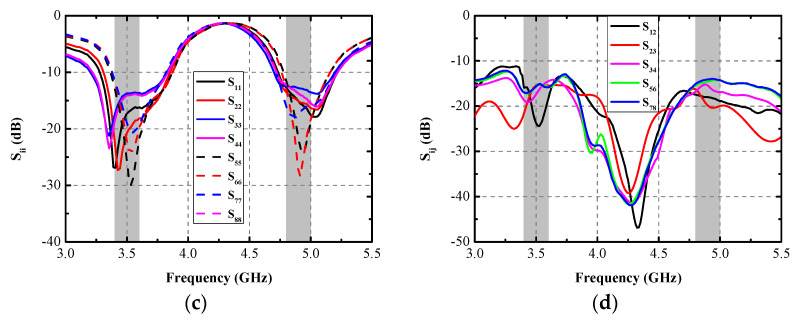
Simulated and measured S-parameters (**a**) S_ii_, (**b**) S_ij_, (**c**) measured S_ii,_ and (**d**) measured S_ij_.

**Figure 11:**
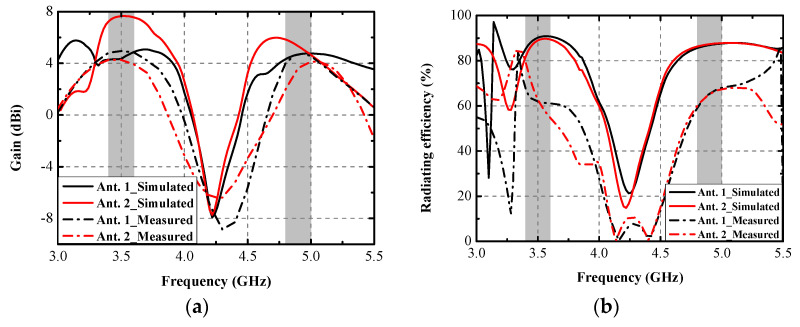
Simulated and measured (**a**) gain and (**b**) radiating efficiency of Ant. 1 and Ant. 2.

**Figure 12:**
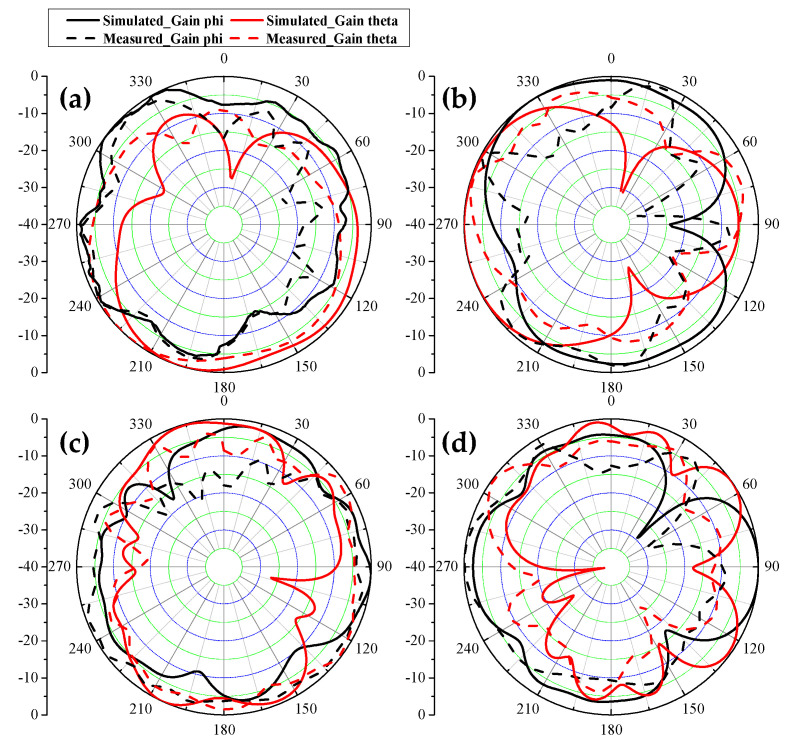
Measured and simulated 2D radiating patterns. (**a**) Ant. 1 is excited at 3.5 GHz, XOY plane, (**b**) Ant. 1 is excited at 3.5 GHz, XOZ plane, (**c**) Ant. 2 is excited at 4.9 GHz, XOY plane, (**d**) Ant. 2 is excited at 4.9 GHz, XOZ plane.

A prototype of the explored antenna was printed and measured to validate the simulated results. Figure 9 presents the photograph of the prototype and test scenarios using a vector network analyzer (VNA: N5224A) and anechoic chamber. In Figure 9a, when Ant. 2 and Ant. 3 are excited, two distinctly resonant modes around 3.5 GHz and 4.9 GHz can be obtained, and excellent uniformity between S_22_ and S_33_ can be observed. Little frequency offset occurs because of the soldering process of the SMA connectors. Figure 9b illustrates the measuring environment of the 2D radiating patterns. Figure 10a,b compare the simulated and measured S-parameters (Sii and Sij, respectively). A slight frequency shift exists between the simulated and measured results, but the measurement can still completely cover the target bands. Measured worst isolation (14.5 dB) of S_23_ appears at around 3.5 GHz. Figure 10c,d present the measured S-parameters of the proposed antenna. All the tested input return loss curves of eight ports can contain the desired bands, and the measured mutual coupling is separately larger than 14.5 dB and 15 dB at 3.5 GHz and 4.9 GHz. Figure 11 provides the simulated and measured gain and radiating efficiency of Ant. 1 and Ant. 2. As shown in Figure 11a, maximum gains of 5 dBi and 4.8 dBi are achieved during the former and latter operating bands, respectively. Radiating efficiency of approximately 60% and 70% is obtained separately at 3.5 GHz and 4.9 GHz, as shown in Figure 11b. The measured and simulated 2D radiating patterns of the proposed antenna are illustrated in Figure 12. The discrepancies between the simulated and measured curves are caused by the soldering process and the installation angle of the antenna when it is tested in the anechoic chamber.

**Figure 13:**
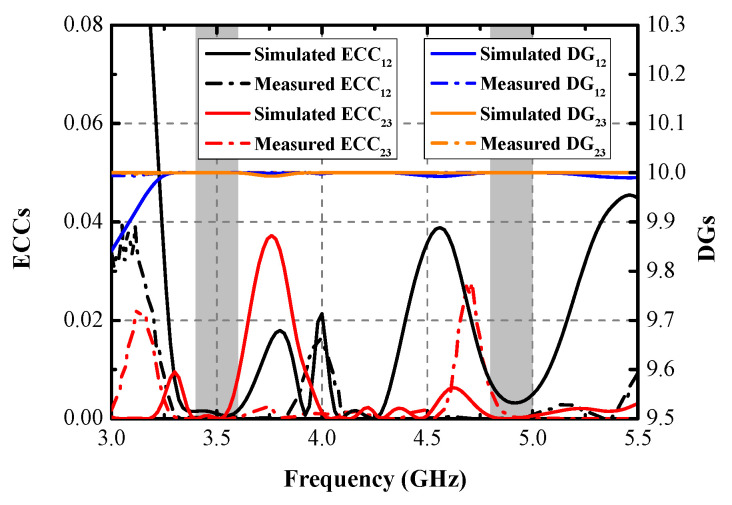
Simulated and measured ECCs and DGs.

**Figure 14:**
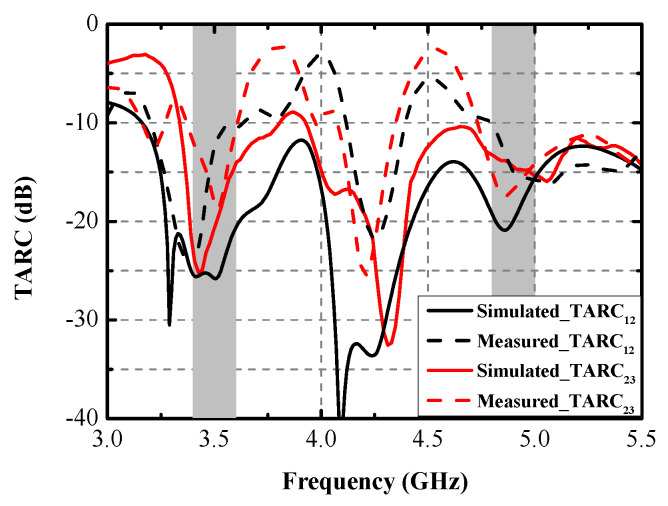
Simulated and measured TARCs.

**Figure 15:**
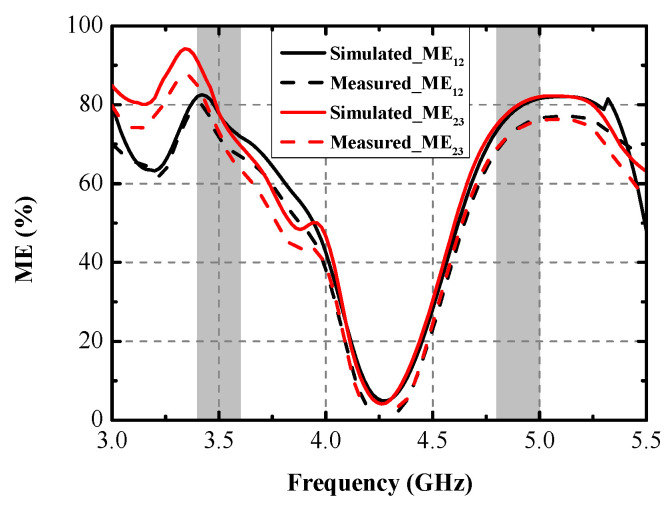
Simulated and measured MEs.

Numerous indicators, including ECC, DG, TARC, and ME, were computed to assess the MIMO performance of the designed MIMO antenna. Figure 13 shows the simulated and measured ECCs and DGs between Ant. 1 and Ant. 2 and between Ant. 2 and Ant. 3. The largest measured ECCs of 0.004 and 0.008 are realized across the former and the latter operating modes, respectively. The ECCs are computed from the radiating results based on Formula (1) [18]. The computed DGs, calculated from Formula (2) [19], are better than 9.99 dB and 9.978 dB within the two target bands, respectively. TARC is the definition of the square root of the ratio of total reflected radio-frequency (RF) power to the total incident power. As shown in Figure 14, the TARC curves are calculated by Equation (3) [20], which are well below the −10 dB level within the two desired bands. ME is defined as the power loss of a realistic antenna in achieving a given power capacity compared with an ideal antenna with total percentage radiation efficiency. ME can be expressed by Equation (4) [21]. Figure 15 compares the simulated and measured ME results between Ant. 1 and Ant. 2, and between Ant. 2 and Ant. 3, respectively. Measured ME values of approximately 70% and 75% are obtained at 3.5 GHz and 4.9 GHz, respectively. Remarkable consistency between the simulated and measured ME curves was observed.

(1) $$\mathrm{ECC}=\frac{{\left|S_{\mathrm{ii}}^{*}S_{\mathrm{ij}}{+S}_{\mathrm{ji}}^{*}S_{\mathrm{jj}}\right|}^{2}}{\left(1-{\left|S_{\mathrm{ii}}\right|}^{2}-{\left|S_{\mathrm{ji}}\right|}^{2})(1-{\left|S_{\mathrm{jj}}\right|}^{2}-{\left|S_{\mathrm{ij}}\right|}^{2}\right)}$$

(2) $$\mathrm{DG}=10\times \sqrt{1-{\mathrm{ECC}}^{2}}$$

(3) $$\mathrm{TARC}=\sqrt{\frac{{{(S}_{11}{+S}_{12})}^{2}{+(S}_{22}{+S}_{21}{)}^{2}}{2}}$$

(4) $$\mathrm{ME}=\sqrt{\eta_{1}\eta_{2}(1-{\mathrm{ECC}}_{12}^{2})}$$

where ƞ_1_ and ƞ_2_ represent the total efficiency of Ant. 1 and Ant. 2, respectively.

Table 2 presents a performance contrast between the presented antenna and other 5G smartphone antennas exploited in recent years. The primary highlights of the proposed antenna are the lowest lateral sideboard, superior isolation performance, and lower ECCs.

## 5. Conclusions

An ultrathin eight-port MIMO antenna functioning in 5G 3.4–3.6 GHz and 4.8–5 GHz is presented in this paper. The explored antenna element obtained a minimized dimension of 9 × 4.2 mm^2^, and the overall volume of the MIMO system was only 145 × 70 × 5 mm^3^. Two kinds of DBs were employed to attenuate the mutual coupling at 3.5 GHz. Besides the design stages of the proposed antenna, the role performed by the DBs were also studied to gain profound understanding of the decoupling mechanism. An application scenario of DHM was given to evaluate the robustness and practicability of the presented antenna. The measured −10 dB impedance band is able to contain the target bands entirely. The measured worst mutual coupling (14.5 dB) appeared in S_23_ around 3.5 GHz. Maximum radiating efficiency of 60% and 75% were obtained within the first and second bands, respectively. The computed results of indicators, such as the ECC (0.008), DG (9.978), TARC (10 dB), and ME (70%), have proved the excellent MIMO performance of the proposed antenna.

## Figures and Tables

**Table 1:** The simulated normalized impedance at two resonant frequencies of each design stage.

Design Evolution	3.5 GHz	4.9 GHz
1st structure	0.094 + 0.518i	0.2946 + 0.9528i
2nd structure	0.5085 + 0.0955i	0.3626 + 0.2061i
3rd structure	0.6245 + 0.0824i	0.3102 + 0.2382i
Final structure	1.08–0.3846i (3.35 GHz) 0.7507–0.3014i (3.5 GHz)	1.3667 + 0.0222i

**Table 2:** Performance contrast between this work and other reported 5G smartphone antennas.

Design	Working Band (GHz)	Total Size (mm^3^)	Dimension of A Single Element (mm^3^)	Decoupling Method	Isolation (dB)	ECC
[14]	3.4–3.6 4.8–5 (−6 dB)	150 × 75 × 7	14.8 × 7 × 0.8	Decoupling structure	15.5 19	0.07 0.06
[15]	3.4–3.6 4.8–5 (−6 dB)	150 × 75 × 7	15 × 7 × 0.8	Neutralization line	11.5	0.08
[16]	3.4–3.6 (−10 dB)	150 × 73 × 6	12 × 4.2 × 0.8	Orthogonal Mode	17	0.06
[22]	3.4–3.6 4.8–5 (−10 dB)	150 × 75 × 6	17.4 × 6 × 0.8	Self-isolated	19.1	0.0125
[23]	3.3–3.6 4.8–5 (−10 dB)	150 × 73 × 7	15.5 × 7 × 0.8	Self-isolated	11	0.15
[24]	4.4–5 (−6 dB)	150 × 80 × 0.787	50 × 30 × 0.787	Shorting pins	18	0.24
[25]	3.4–3.6 (−10 dB)	150 × 75 × 5.3	16.1 × 4.5	Self-isolated	20	0.4
This work	3.4–3.6 4.8–5 (−10 dB)	145 × 70 × 5	9 × 4.2 × 0.8	DBs	14.5 15	0.004 0.008

## Data Availability

Not applicable.
